# *Bambi* and *Sp8* Expression Mark Digit Tips and Their Absence Shows That Chick Wing Digits 2 and 3 Are Truncated

**DOI:** 10.1371/journal.pone.0052781

**Published:** 2012-12-28

**Authors:** Jesús C. Casanova, Claudio Badia-Careaga, Verónica Uribe, Juan José Sanz-Ezquerro

**Affiliations:** 1 Department of Cardiovascular Development and Repair, Centro Nacional de Investigaciones Cardiovasculares, CNIC, Madrid, Spain; 2 Department of Immunology and Oncology, Centro Nacional de Biotecnologia, CSIC, Madrid, Spain; 3 Department of Molecular and Cellular Biology, Centro Nacional de Biotecnologia, CSIC, Madrid, Spain; Instituto Gulbenkian de Ciência, Portugal

## Abstract

An often overlooked aspect of digit development is the special nature of the terminal phalanx, a specialized structure with characteristics distinct from other phalanges, for example the presence of ectodermal derivatives such as nails and claws. Here, we describe the unique ossification pattern of distal phalanges and characteristic gene expression in the digit tips of chick and duck embryos. Our results show that the distal phalanx of chick wing digit 1 is a genuine tip with a characteristic ossification pattern and expression of *Bambi* and *Sp8*; however, the terminal phalanx of digits 2* and 3 is not a genuine tip, and these are therefore truncated digits. *Bambi* and *Sp8* expression in the chick wing provides a direct molecular assessment of digit identity changes after experimental manipulations of digit primordia. In contrast, digits 1 and 2 of the duck wing both possess true tips. Although chick wing-tip development was not rescued by application of Fgf8, this treatment induced the development of extra phalanges. Grafting experiments show that competence for tip formation, including nails, is latent in the interdigital tissue. Our results deepen understanding of the mechanisms of digit tip formation, highlighting its developmental autonomy and modular nature, with implications for digit reduction or loss during evolution. * Numbering of wing digits is 1, 2, 3 from anterior to posterior.

## Introduction

The vertebrate limb is a classic model for the study of pattern formation and morphogenesis during embryonic development (reviewed in [1]). Limbs arise from the lateral plate mesoderm as small buds composed of undifferentiated mesenchyme encased in an ectodermal jacket. Outgrowth of the limb bud in the proximo-distal axis is directed by a signalling centre, the apical ectodermal ridge (AER), a thickened specialized epithelium at the dorso-ventral boundary of the distal limb bud (reviewed in [2]). AER function is mediated by members of the fibroblast growth factor family, particularly Fgf8. As the limb bud grows, skeletal elements progressively differentiate. Initial mesenchymal condensations give rise to a cartilage template that is later replaced by bone through endochondral ossification [3]. The sequence of cartilage condensation follows a proximo-distal order, with elements near to the body (humerus/femur) appearing first, intermediate elements (ulna-radius-fibula) next and digits forming last.

Digits are one of the most diversified anatomical structures in tetrapods as a result of their evolutionary adaptation to a wide range of specific functions. The number and shape of digits varies greatly across animal groups, and even digits of a same limb can appear quite different. Digit identity is defined by a set of characteristic morphological traits (length, number and shape of phalanges) that are specific to its position in the antero-posterior axis of the autopod. There has been intense debate as to whether this identity is determined by a unique molecular code established by morphogens and fixed early by antero-posterior patterning pathways, or imposed later onto initially equivalent condensations by local signalling cues from adjacent tissues. Late determination would imply a degree of plasticity in the final identity of digit primordia, and is supported by the successful experimental alteration of the number of phalanges by surgical manipulation or application of signalling molecules at stages where condensations have already formed [4], [5]. The translation of early AP information is thought to occur in the interdigital mesenchyme, which would thus confer identity on the digital primordia [4], [6].

Accurate assessment of digit identity is particularly important in patterning studies of developing limbs, where transformations, duplications or loss of digits occur. This assessment can be difficult in the mouse, since the morphology of the three central digits is similar [7]. This is not the case in chick, which is one reason for its wide use for this type of study [8]. Each of the four toes in the chick leg (I-IV) has a different number of phalanges, making their identification straightforward. The wing retains only three digits (1, 2, 3) and the morphology of each is quite distinct, similarly enabling an easy identification [9].

An often overlooked aspect of digit anatomy is the distinctive nature of the terminal phalanx [10]. We have characterised the ossification pattern and gene expression in digit tips of chick and duck embryos and found that digits 2 and 3 of the chick wing do not have a true tip and are thus truncated digits, contrasting with the last phalanx of digit 1, which is a true tip. We used the unique expression of *Bambi* in tips to confirm the identity of transformed chick wing digits after experimental manipulations of digit primordia. Although tip formation was not rescued by the sole application of Fgf8, this treatment induced extra phalanges in digits 1 and 3.

## Results

### Chick Wing Digits 2 and 3 do not have a True Tip and are thus Truncated Digits

**Figure 1:**
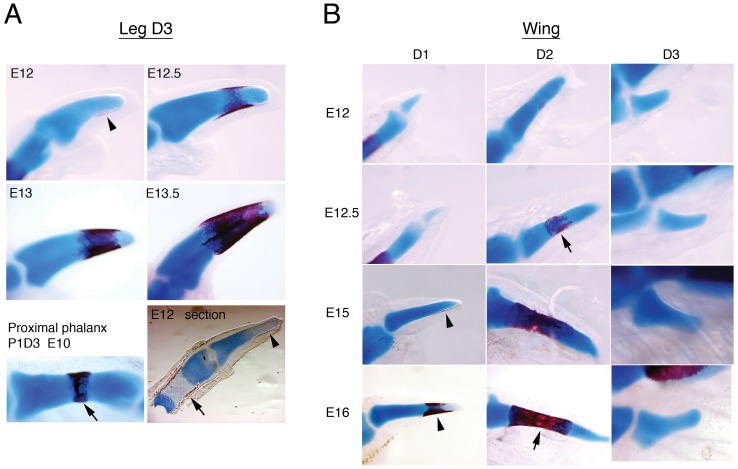
Ossification pattern of the terminal phalanx in chick digits. A: Ossification in the tip of leg digit 3 on the indicated days of development, as detected by alizarin red staining. Note the ventral initiation of ossification (arrowhead) and the progression towards the distal end. A proximal phalanx (phalanx 1 of digit 3, P1D3, at E10), with an evident central ossification ring, is shown for comparison (arrows). The bottom right panel shows a stained longitudinal section in which alizarin red is evident at the distal extreme of the tip phalanx; other panels show whole-mount specimens. B: Ossification of the terminal phalanges of chick wing digits. Ossification in digit 1 is similar to that in leg tips, starting ventrally and progressing distally (arrowheads). In contrast, the ossification of digit 2 resembles a proximal/intermediate phalange, with the presence of a central ossification ring (arrows).

To characterise the special features of digit tips, we analysed the ossification pattern of phalanges in chick embryos. Long bones such as phalanges have an endochondral mode of ossification, in which a cartilage template is laid first and then substituted by bone. Ossification starts in the centre of the mature cartilage, where hypertrophic chondrocytes die and are replaced by osteoblasts, forming the primary ossification centre. In parallel, a bone collar is formed in the perichondrium. We observed this pattern in all leg phalanges (Figure 1A, arrows) except the terminal ones, where a distal alizarin-red stained area was observed, appearing first ventrally and eventually covering the very end of the phalanx (Figure 1A, arrowheads). The ossification pattern thus highlights another distinctive characteristic of terminal phalanges. In the wing, this distal ossification pattern occurs in the terminal phalanx of digit 1 (Figure 1B, arrowheads), but the last phalanx of digit 2 shows an ossification pattern comparable to proximal and middle phalanges, with a central ossification ring (Figure 1B, arrows). No ossification was detected in the digit 3 phalanx during the period analysed (up to E20 corresponding to in ovo development; this elements ossifies pot-hatching [11]).

**Figure 2:**
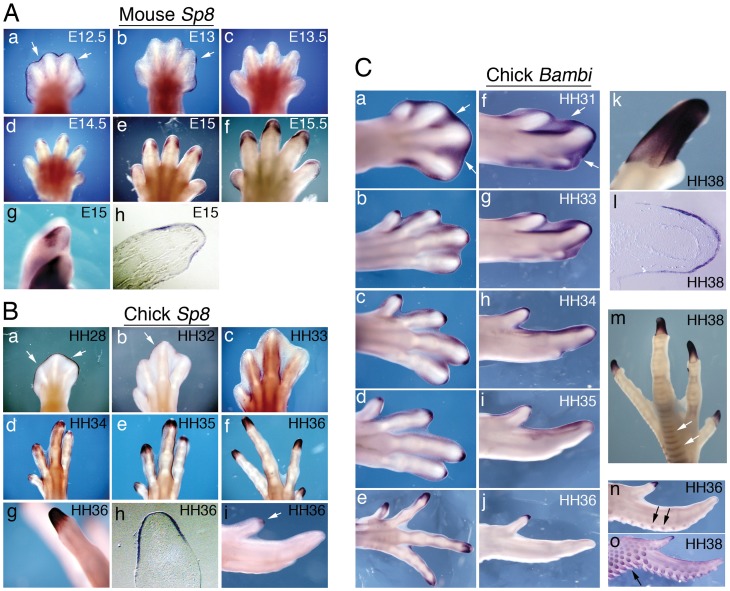
Expression of *Sp8* and *Bambi* in digit tips. A: *Sp8* in situ hybridization in mouse digits at the indicated developmental stages. (a-g) Whole-mount hind limbs. Note that *Sp8* is expressed in the AER up to E13 (a,b, arrows) and later reappears in the tip ectoderm (d-f). Expression is stronger dorsally (g) and examination of stained paraffin sections shows that it is restricted to the ectoderm (h). B: In situ hybridization for *Sp8* in chick digits at the indicated Hamburger and Hamilton stages. *Sp8* is expressed in the AER of all leg digits up to HH32 (a,b, arrows) and later reappears in the tips of digits (d-f). Expression is limited to the tip, showing a sharp boundary (g), and is restricted to the ectoderm (h, paraffin section). (i) In the wing, *Sp8* is expressed at the tip of digit 1 (arrow) but is absent from the ends of digits 2 and 3. C: *Bambi* in situ hybridisation in chick digits at the indicated stages (a-e, legs; f-j, wings). Note the strong initial expression in the interdigital space mesenchyme (a, f, arrows) that subsequently fades away, and the expression in the digit tips as the last phalanx forms. This terminal expression is observed in all toes (e) but is only present in the tip of digit 1 in the wing (j). Within digits, *Bambi* is sharply restricted to the tip (k) and is expressed only in the ectoderm (l, paraffin section). *Bambi* is also expressed (arrows) in leg scales (m) and feather buds (n,o).

**Figure 3:**
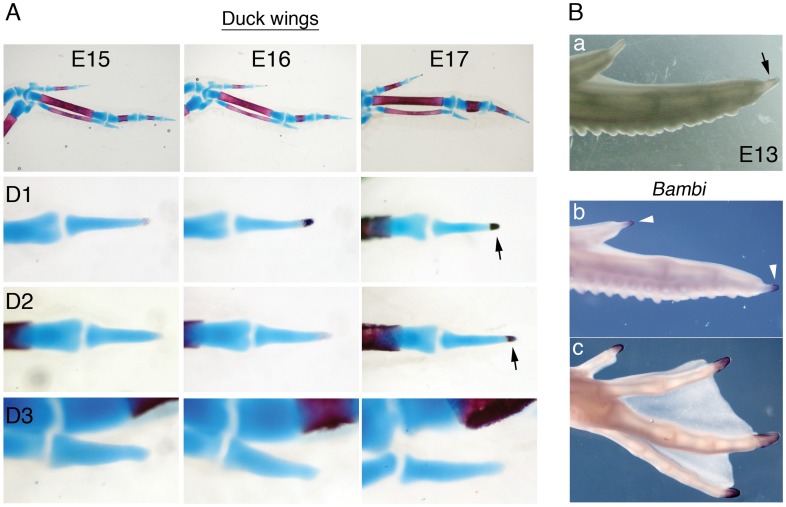
Digit 2 of the duck wing has a genuine tip. A: The pattern of ossification (alizarin red staining) of the last phalanx of digits 1 and 2 is distal (arrows). B: (a) Fresh wing specimen, showing the evident claw at the end of digit 2 (arrow). (b,c) In situ hybridisation showing *Bambi* expression at the tips of duck digits at E13. (b) Expression is detected in wing digits 1 and 2 (arrowheads). (c) In the leg, *Bambi* is expressed in all toe tips.

Several genes are expressed specifically in the tip and not in proximal or middle phalanges [10]. To identify other genes associated with digit tips and to further investigate the difference between the distal phalanges of digits 1 and 2, we analysed the later expression of genes that are expressed distally early in limb development. One of these, *Sp8*, is a transcription factor whose function is essential for the maintenance of the AER [12], [13]. *Sp8* is expressed in the AER of mouse and chick limb buds until its regression at digit formation stages, and its expression is no longer detected from E13.5 in mouse (Figure 2A,c) and HH33 in chick (Figure 2B,c) [14]. Interestingly, we found that *Sp8* expression reappears later in the distal ectoderm covering the distal-most phalanges, both in mouse (from E14.5, Figure 2A,d–f) and chick legs (from HH34, Figure 2B,d–f). This expression forms a sharp boundary between the penultimate and terminal phalanx (Figure 2A,g, mouse and Figure 2B,g, chick) and is restricted to the ectoderm, as confirmed in sections (Figure 2A,h, mouse and Figure 3B,h, chick).

We also analysed expression of *Bambi*, a membrane-bound pseudo-receptor that inhibits the Bmp signalling pathway [15]. During mouse limb development *Bambi* is expressed in the AER and subjacent mesenchyme of the limb buds, and later in the interdigital mesenchyme, the perichondrium and the digit tips [16]. During digit formation in chick, *Bambi* expression is high in the interdigital mesenchyme (Figure 2C,a,f; [17]) and is also present in digit tips (Figure 2C,e,j; [10]). This tip expression is very prominent and, as in mouse, is restricted to the ectoderm of the terminal phalanx (Figure 2C,k–l, [16]). We also detected *Bambi* expression in other ectodermal derivatives such as scales (Figure 2C,m) and feather buds (Figure 2C,n–o; Figure S1F).

The above observations suggest that *Sp8* and *Bambi* are molecular markers of digit tips. In the chick wing, although *Sp8* and *Bambi* are both strongly expressed at the tip of digit 1, expression is absent from the distal region of digits 2 and 3, as can be seen by in situ hybridisation in whole mount samples (Figure 2B,i; 2C,j; [10]) and on sections (Figure S1A–D) and confirmed by real time quantitative PCR (Figure S1E). Moreover, digits 2 and 3 apparently do not possess a tip, based on their morphology and lack of claws.

To confirm the association of *Bambi* and *Sp8* expression with the presence of a bona-fide digit tip, we performed experiments to induce digit truncation by local application of noggin, a BMP inhibitor, as previously described [18]. Beads soaked in noggin were applied at stage HH29-31 to the distal region of the primordia of digits that bear a clear tip and normally show expression of *Bambi* and *Sp8* (wing digit 1 and leg digit 3). Embryos were collected 4–5 days after the operation and expression of *Bambi* or *Sp8* analysed by whole-mount in situ hybridisation. Application of noggin resulted in truncated digits that lacked both the tip and expression of the markers (23/23 cases for *Bambi* and 2/2 cases for *Sp8* in the wing, Figure S2A,E; 15/15 cases for *Bambi* in the leg, Figure S2C). Control beads did not produce any effect on digit formation or gene expression (5/5 cases for *Bambi* in wing and leg, Figure S2B,D). These results indicate that loss of the tip (truncation) implies loss of *Bambi* and *Sp8* expression.

The absence of expression of *Bambi* and *Sp8*, together with the different ossification pattern, thus strongly suggests that the distal-most phalanges of digits 2 and 3 in the chicken wing are not true tips and that these digits are truncated.

### The Terminal Phalanx of Duck Wing Digit 2 is a True Tip

To determine if this truncation is a general feature of avian wing digits, we analysed ossification and *Bambi* expression in duck embryo digits (Figure 3). All the terminal phalanges of duck toes have the typical distal ossification centre of digit tips (Figure S3A). The duck wing has three digits but, unlike chickens, digit 2 has three phalanges. In the duck wing, a distal ossification pattern was observed in the distal-most phalanx not only in digit 1, but also digit 2 (Figure 3A, arrows; as in chick, ossification was not detected in digit 3 in the embryonic period examined). Moreover, in whole embryos at day 13, the end of wing digit 2 has a tip appearance, with a pointed claw-like structure (Figure 3B,a, arrow). Analysis of *Bambi* confirmed expression in the distal-most phalanx of all leg digits and also of wing digits 1 and 2 (Figure 3B,b–c, arrowheads). Thus, unlike the situation in chick, the final phalanx of digit 2 in the duck wing is a genuine tip. Also, *Bambi* expression was observed in duck feather buds, scales and perichondrium as in chicken (Figure S5).

In mammals, despite the general proximal-to-distal sequence of limb element formation, the terminal phalanges ossify before the middle ones [19]. To characterize osteogenesis in avian limb digits, we re-analysed the sequence of phalanx ossification in chick [11], [20] and compared it with the sequence in duck, which has not been reported previously. Our results (Figures S3, S4 and Tables 1–4 in File S1) show that, with the exception of foot digit 4, distal phalanges in chick legs and wings ossify last. In the duck leg, distal phalanges ossify before middle ones in all toes (except for toe 2, in which the terminal phalanx ossifies just after the middle one). Thus while the phalanx ossification sequence in chick legs differs from that in the mouse, the sequence in duck legs is more similar to that observed in mouse than in chick. In the duck wing, however, the distal-most phalanges of digits 1 and 2 ossify after the proximal and middle phalanges, similar to chick.

### Tip Expression of *Bambi* Confirms Altered Digit Identity in the Chick Wing

**Figure 4:**
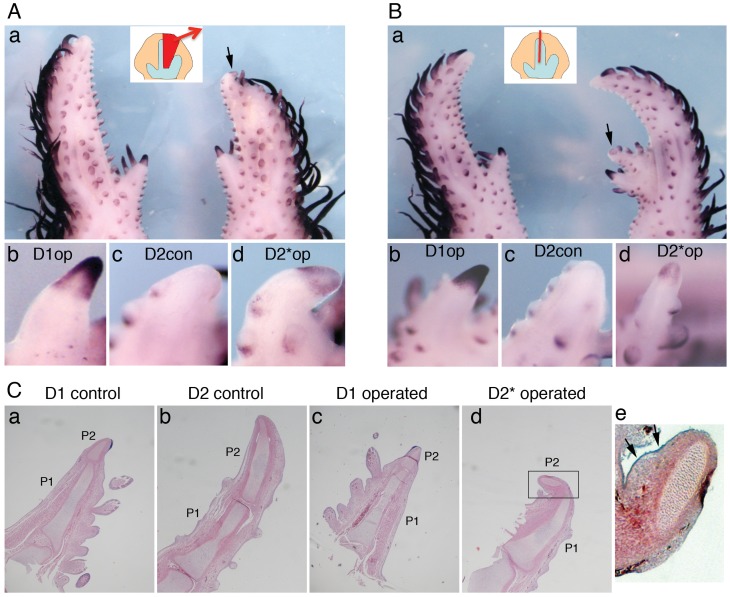
*Bambi* tip expression marks changes of digit identity triggered by surgical manipulations. Two surgical manipulations were performed in HH27 wings to transform the identity of digit 2 towards digit 1. Seven days after the operation, *Bambi* expression was detected by in situ hybridisation. A: In type I experiments, the posterior part of the digit 2 primordium and the posterior interdigital space 2 were removed (see scheme in the inset in a). (a) The remnant digit 2 primordium has developed into a shorter digit (transformed digit 2*) with a tip positive for *Bambi* expression (arrow). (b) Magnified view of *Bambi* expression in the tip of digit 1 in the operated limb. (c) *Bambi*-negative tip of digit 2 in the control (non-operated) wing. (d) *Bambi* expression in the tip of the operated and transformed digit 2*. B: In type II experiments the digit 2 primordium was bisected (see scheme in the inset in a). (a) The extra digit formed from the anterior half of the digit 2 primordium (transformed digit 2*) shows expression of *Bambi* in the tip (arrow). (b) Magnified view of *Bambi* expression in the tip of digit 1 in the operated limb. (c) *Bambi*-negative tip of digit 2 in the control wing. (d) *Bambi* expression in the tip of the operated and transformed digit 2*. C: Digits from the specimen in A (and additionally the digit 1 from the control wing) were sectioned after the *Bambi* in situ hybridisation and stained with haematoxylin & eosin to detect transformation of cartilage elements. The length of phalanx 2 (P2) relative to phalanx 1 (P1) in transformed digit 2* is reduced, resembling the proportions in digit 1. (e) Magnified view of the boxed area in d (rotated 90°). Note the expression of *Bambi* in the tip ectoderm of the transformed digit 2* (arrows).

**Figure 5:**
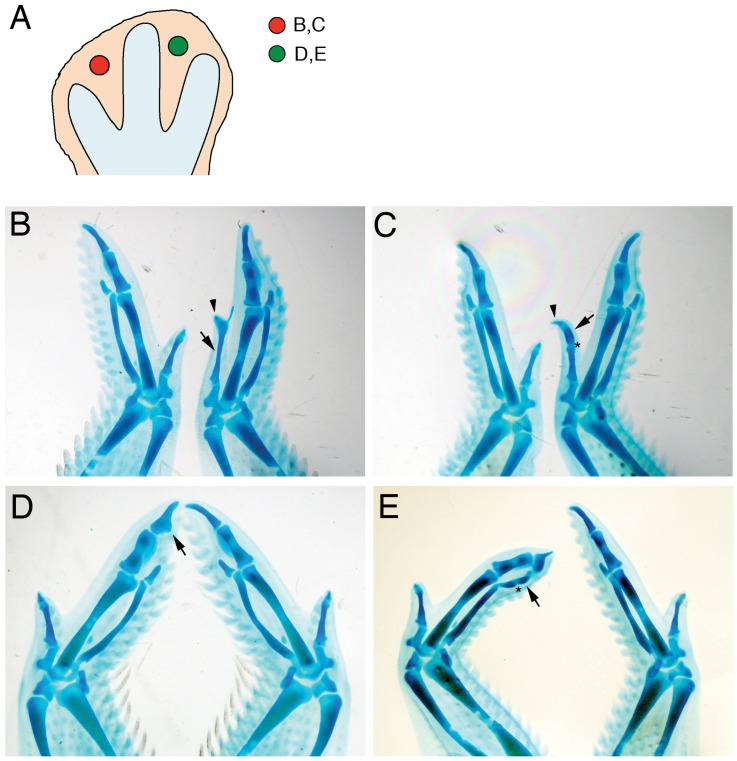
Fgf8 induces elongation and extra phalanges in digits 1 and 3, but not digit 2, of the chick wing. Beads soaked in Fgf8 (1 mg/ml) were applied to the first (B,C) or second (D,E) interdigital spaces of HH27 wings. Five days after the operation embryos were collected and stained with alcian green to reveal skeletal elements. A: Scheme showing the position of the beads at the time of operation. Text refers to the panels showing each experiment. B, C: Application to the first interdigital space induced elongation of digit 1 (B, arrow), sometimes with the production of a complete extra phalange (C, arrow) with a new joint (asterisk). In both cases, the tip is normal (arrowheads). D: Application to the second interdigital space did not induce elongation of digit 2, but caused widening (D, arrow). E: In contrast, this treatment induced elongation of digit 3 with the formation of an extra phalanx (arrow) with a new joint (asterisk). Operated wings are shown on the right in panels B,C and on the left in panels D,E (all dorsal views).

Experimental manipulations of digit primordia show that the specification of digit identity is labile until digit condensation stages and that final identity is imposed by local cues from the posterior interdigital (postID) mesenchyme [4], [21]. Experiments by Dahn and Fallon [4] were mostly performed in the leg, and digit transformations in the wing were induced only by removing the postID; moreover, digit transformation was assessed by differential morphology alone, based on the relative lengths of the phalanges of digits 1 and 2. To assess the effect of experimental digit transformations in the wing with molecular markers, we analysed expression of *Bambi* and *Sp8* after two surgical manipulations that transform wing digit 2 into digit 1, which would therefore be predicted to acquire a tip. Results are shown in Figure 4, Figure S6 and Tables 5–6 in File S2. In the first manipulation (experiment type I), removal of the posterior half of the digit 2 primordium and the postID2 transformed the remaining anterior half of digit 2 into a shorter digit in 60/95 (63%) of cases. In the second manipulation (experiment type II), bisection of the digit 2 primordium transformed the anterior portion into a shorter digit in 29/71 (41%) of cases. Of the morphologically transformed digits from type I experiments, 8/54 (15%) expressed *Bambi* at their termini (Figure 4A) and 1/6 (17%) expressed *Sp8* (Figure S6A). Of the morphologically transformed digits from type II experiments, 6/18 (33%) expressed *Bambi* at their tips (Figure 5B, Figure S6C) and 3/11 (27%) expressed *Sp8* (Figure S6B,D). Analysis of paraffin sections confirmed the presence of a transformed distal phalanx (Figure 4C) with the typical appearance of a terminal phalanx, including the absence of a central ossification centre (Figure 4C,d) and expression of *Bambi* in the tip (Figure 4C,e, arrows). These results provide the first molecular criterion for assessing altered digit identity after experimental manipulations of digit primordia in the chicken wing.

### Fgf8 can Elongate and Induce Extra Phalanges in Chick Wing Digits 1 and 3, but not Digit 2

The observation that digit 2 in the chicken wing is a truncated digit prompted us to explore the possibility of rescuing the tip. Since Fgf8 has been shown to induce digit elongation [5], we applied Fgf8-soaked beads to the second ID of the chick wing at stage HH26-27 (Figure 5, Figure S7). To observe the effects in a digit that has a true tip, in control experiments beads were implanted into the first ID. Embryos were collected 4 to 7 days after the operation, and stained with alcian green to reveal skeletal elements. Application to the first ID produced a clear elongation of digit 1, with the tip phalanx unaltered, in 12/23 cases (Figure 5 B,C; Figure S7A). In only 3/23 cases an extra cartilage element fused to digit 1 was observed (Figure S7B), as previously reported [22]. The degree of elongation was variable, ranging from a longer first phalanx (Figure 5B) to the formation in two cases of a complete extra phalanx (with an extra joint), resulting in a three-phalange digit (Figure 5C). In contrast, application of Fgf8 to the second ID did not elongate digit 2 in any of 42 cases. Instead, widening of phalanges was observed in 30/42 cases (Figure 5D), and in some cases (n = 11/42) an extra cartilage nodule formed next to the interphalangeal joint (Figure S7C). Interestingly, the same treatment induced elongation of digit 3 phalanx in 13/42 cases (Figure 5E, Figure S7D,E) and segmentation and formation of a new phalanx was indeed seen in 6 cases (Figure 5E, Figure S7E). These results indicate that, whereas Fgf8 can induce extra phalanges in digit 1 and digit 3, it is unable to elongate digit 2 and furthermore cannot rescue the absent tip in any case.

### Interdigital Distal Tissue can give Rise to Tips with Claws

**Figure 6:**
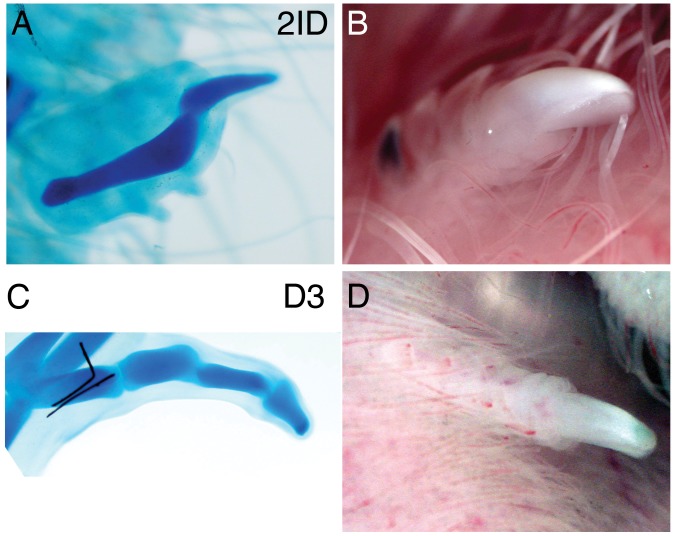
Interdigital tissue can form ectopic digits with tips and claws. Explants of distal interdigital space 2 (2ID: A,B) or distal digital ray 3 (D3: C,D) were taken from HH27 legs (distal 150 µm plus AER) and grafted to the somites of HH20 hosts. 2ID and D3 explants both formed ectopic digits with phalanges (alcian blue staining, A,C) and claws (white keratinized area in fresh specimens, B,D), detected 8–10 days after transplantation.

Although the interdigital mesenchyme is normally eliminated by apoptosis, it can form extra digits in situ when exposed to chondrogenic signals ([10] and referecnes therein). Although these extra digits often include a terminal phalanx, the potential of interdigital ectoderm to contribute to tip structures has not been determined before. To test this potential, we grafted the distal-most tissue, including the AER, of leg interdigital spaces 3 (ID3) and 2 (ID2) from stage HH27-28 embryos to the somites of stage HH20-22 recipients. As controls, we grafted equivalent tissue from the tip of digit 3. Outgrowth of the grafts was readily observed 2 to 4 days after the operation. Examination of embryos nine to ten days after transplantation revealed ectopic digits derived from the grafted tissue (n = 12/18 for interdigital tissue, n = 8/9 for digit 3 tips, Figure 6). Alcian blue staining revealed a typical acuminate distal phalanx structure (see Figure 6A for ID2, Fig. 6C for digit 3 tip), and also the presence of, keratinized claws (Figure 6B for ID2, Fig. 6D for digit 3 tip). These results show that the distal interdigital tissue is competent by itself to form digit-like structures when removed from its position and grafted to ectopic sites in vivo. Moreover the graft is capable not only of generating cartilage from the mesenchyme but also of producing ectodermal derivatives.

## Discussion

Our results identify molecular and histological features characteristic of terminal phalanges. Taken together with previous data, these features allow a true digit tip to be defined as a distal phalanx with a pointed shape, a distal ossification centre, expression of *Bambi, Sp8* and other genes, and containing ectodermal derivatives such as nails or claws. The inclusion of molecular features as well as morphological criteria is particularly important for assessing distal phalanx identity at embryonic stages, when an acuminate shape is an insufficient criterion for identifying a true tip phalanx. Comparative analysis of these features in chick and duck shows unequivocally that digits 2 and 3 in the chicken wing lack a genuine tip and are thus truncated digits.

Clawed tips are surprisingly common in the wings of extant birds. Digit 1 claws are generally present in ratites, galliforms, anseriforms, ciconiiforms and charadriiforms [23]. Digit 2 claws are rarer, but are found in some anseriforms and are very conspicuous in opisthocomiforms. This, together with paleontological data from extinct birds, suggests that tips are an ancestral character, operating as an independent evolutionary module that is selectively maintained or lost as a result of functional adaptations (for instance retention for tree climbing and elimination for flight optimization) or retained due to a lack of selective pressure. Moreover, the lack of a true tip in chick wing digit 2 highlights the derived nature of this particular limb and indicates a need for caution when using it as a reference model of digit morphogenesis.

An interesting observation is the expression in the tip ectoderm of *Bambi* and *Sp8*, which reappear after having been switched off in the AER. This restricted expression could be related to the differentiation of the tip ectodermal derivatives. However, no phenotype in the nails (or digits) has been reported for Bambi knock-out mice, which are viable [24]. This suggests that expression of *Bambi* is not necessary for tip or nail formation, despite being a marker associated with these structures. Bambi could be involved in subtle and as yet uncharacterised nail homeostatic mechanisms. Alternatively, compensatory mechanisms might operate in its absence to generate a normal tip. On the other hand, Sp8-null mice lack digits, which prevents the analysis of its functions in the tip.

The specific expression of genes in the tip, both in mesenchyme and ectoderm, supports the existence of a molecular tip programme for the development of its special features. These include the distal ossification pattern, the special microvasculature, the development of nails and claws, and the regeneration potential, unique among the organs of higher vertebrates [25], [26]. Understanding the gene regulatory networks defining this tip programme is essential for any attempt to improve the regenerative capability of digits (for instance after proximal amputations). The recent finding that digit tip regeneration is driven by lineage-restricted resident stem cells [27], [28] suggests the existence of a local niche required by these progenitors. It will be interesting to define how and when this niche is established, and another as yet uncharacterized feature of the tip may be the expression of stem/progenitor cell markers. Interestingly, our grafting experiments show that competence to generate the distal-most digit structures, both mesenchymal and ectodermal, is latent in the interdigital spaces, but is actively blocked in these regions during normal development.

The molecular characterization of a true tip enables a more precise definition of digit identity, providing a new diagnostic feature in addition to the number, size and shape of phalanges. This has allowed us to confirm transformations of digit identity in the chick wing, which were not fully explored previously. Our results show that digit 2 acquires expression of *Bambi* and a tip either after bisection of its primordium (an experiment not reported before in wing) or after elimination of the posterior interdigital space. These results strongly support the possibility of fully transforming the identity of a digit by activating the tip programme, and show that digit transformation involves not only changes in phalanx number but also functional transformations. The low frequency of complete transformations (15–33%) probably reflects the narrow window of plasticity before identity is irreversibly fixed, as well as the technical limits to the precision of these surgical manipulations. Molecular identification of digit tips will also be useful for assessing digit transformations induced by genetic manipulations and those resulting from evolutionary adaptations, including digit truncations.

The production of additional phalanges in a digit by the sole application of Fgf8 reinforces the central role of Fgf signalling in digit growth and patterning. This had only been attained before by application of hedgehog signals [4], [5], [22], while Bmps, proposed as determiners of digit identity, are ineffective [6]. Application of Fgf8 to the first ID at slightly earlier times (HH26) led to the formation of extra cartilage and fusion between digits 1 and 2, as had previously been reported [22]. The timing and position of bead application thus seems to be critical, since quite different phenotypes can be obtained by the same signal (see [5], [29].

Remarkably, Fgf8 induced elongation and extra phalanges in wing digits 1 and 3, but not digit 2. The molecular basis for this differential competence is not known, but it is interesting that responsiveness resides in the anterior and posterior-most digits in the wing. The non-responsiveness of digit 2 to Fgf signalling might be related to its special transcriptional profile, which is different from all other digits [30]. Fgf8 did not rescue the tip in the digits lacking it, even in digit 3, where elongation and extra phalange induction was effective. This suggests that maintenance of growth by Fgf signalling is not sufficient to establish the tip programme, which requires other, independent inputs.

Our findings provide a framework for investigating the mechanisms operating during normal digit development (digit elongation, phalanx formation, cessation of growth and tip induction) and also the aetiology of congenital human malformations affecting the digits [31]. Moreover, the regulation of the tip programme (its activation and timing) is fundamental to the understanding of digit evolution, particularly in cases where digits have been shortened or lost.

## Materials and Methods

### Digit Nomenclature

Wing digits are numbered 1,2,3 from anterior to posterior, following recent analysis of identity by fate mapping and transcriptomic profiles [30], [32], [33].

### Embryos

Fertilised chick eggs (Rhode Island Red) were obtained from Granja Santa Isabel (Cordoba, Spain) and fertilised duck eggs (Peking and Campbell cross) were obtained from Granja San Fernando (Somo, Spain). Eggs were incubated at 38°C. Chick embryos were staged according to [34] and duck embryos according to [35]. Mouse embryos were collected at the indicated gestational stage (E0.5 was defined as noon of the day on which a vaginal plug was detected).

All animal work has been performed according to the guidelines of the Committee on Ethics and Animal Welfare established at CNIC, in accordance to Spanish laws. The CNIC Committee on Ethics and Animal Welfare has approved this study.

### Alcian Blue/alizarin Red Staining

Embryos were fixed in 95% ethanol and stained for cartilage and bone according to standard protocols. The earliest time of positive alizarin red staining for a given phalanx was recorded as the ossification stage in Tables 1–4 in File S1. For each stage described, at least 3 specimens were analysed (n = 3 to 9) of which at least 2 showed ossification at the indicated stage (n = 2 to 9).

### In situ Hybridisation

Embryos were fixed in 4% PFA. In situ hybridizations in whole mount embryos or in 7 µm paraffin sections of HH36 chick wing digits were done using standard protocols [36], [37]. Probes used were chick and mouse Sp8 [38] and chick Bambi from sequence EST 603482731F1 of the Manchester chick database [39]. The chicken probes were also used for duck specimens.

### Surgical Manipulations

The protocol described in [4] was used. Wings of stage HH26-28 chicken embryos were exposed and the posterior part of the digit 2 primordium plus the posterior interdigital space were removed with the aid of tungsten needles and scissors (experiment type I) or the digit 2 primordia was bisected with microsurgery scissors (experiment type II). At least six independent experiments were performed for each type of experiment. Embryos were fixed and processed 5–7 days after the operation.

### Interdigital Tissue Grafts

Distal tissue (150 µm mesenchyme plus the overlying AER) was dissected from HH27-28 legs. Pieces from digit ray 2 or 3, or interdigital space 2 or 3 were independently grafted to the somites of HH20-22 host embryos. After incubation for 9–10 days embryos were photographed and analysed for the presence of ectopic digits and claws, and processed for cartilage staining.

### Application of Fgf8 and Noggin Beads

Heparin-acrylic beads (Sigma) were soaked for 1 hour at room temperature in recombinant mouse FGF8 (1 mg/ml, R&D Systems) or recombinant mouse noggin (1 mg/ml, R&D Systems). FGF8-soaked beads were implanted into stage HH26-28 interdigital spaces 1 and 2 of the wing limb bud. Noggin-soaked beads were placed in the tip of digit primordia (digit 1 in the wing and digit 3 in the leg) at stages HH28-31. Embryos were collected 3–6 days after implantation and fixed in 4% PFA for in situ hybridisation and in 5% TCA for Alcian Green cartilage staining.

### Real Time Quantitative PCR (Q-PCR)

Tips from wing digits 1 and 2 were collected with micro-dissection scissors from chick embryos at stage HH36 (10 days of incubation). Tips were immediately frozen in dry ice. Each sample (digit 1 tips, digit 2 tips) consisted of a pool of 30 tips coming from 15 limbs, taking right and left limbs. Three independent samples were taken per digit tip. RNA was extracted with the RNAeasy extraction kit (Quiagen) and cDNA synthesised using the High Capacity cDNA Reverse Transcription Kit (Applied Biosystems). Quantitative PCR was conducted in an AB 7900-FAST-384 thermal cycler (Applied Biosystems) using SYBR green. Each sample was amplified in triplicate. GAPDH and HPRT were used as normalization controls. Data analysis was performed using the Biogazelle qbasePLUS program, setting the target scaling to the minimum value. Relative expression results are given as the normalized relative quantity +/− SEM in arbitrary units in a linear scale. Statistical significance was assessed using the Student’s t test (p values: * p<0.05, *** p<0.001).

Primers used were:

GAPDH:

Forward: 5′-CCATTCCTCCACCTTTGATG-3′

Reverse: 5′-ACCAGGAAACAAGCTTGACG-3′

HPRT:

Forward: 5′-TGACAAGTCAATCCCCATGA-3′

Reverse: 5′-AACTGGCCACTTTCACCATC-3′

BAMBI:

Forward: 5′-TGGTGCTCCTTATCATGCTG-3′

Reverse: 5′-TTGCCACCTGTCCTTTCTTC-3′

SP8:

Forward: 5′-TGCCACGAGAAAGCATTACC-3′

Reverse: 5′-AGCTTTGATGGACGCTTCAG-3′

## Supporting Information

Figure S1 *Bambi* and *Sp8* expression in the chick wing digits 1 and 2 tips. A–D, F. In situ hybridisation on tissue sections from stage HH36 wings. A: Strong expression of *Bambi* is observed in the digit 1 tip ectoderm (arrow). Expression is also seen in the perichondrium (pc). B: Although *Bambi* expression is observed in the perichondrium (pc) and feather buds (fb), it is absent from the tip of digit 2 (arrowhead). C: *Sp8* is strongly and specifically expressed in the digit 1 tip ectoderm (arrow) but is absent from digit 2 tip (arrowhead in D). E: Real time quantitative PCR (Q-PCR) analysis of the expression of *Bambi* and *Sp8* in digits 1 and 2 tips. A significantly higher expression of both markers is observed in digit 1 tips than in digit 2 tips. Results are given as the normalized relative quantity (+/− SEM) in arbitrary units (*: p<0.05, ***: p<0.001 Student’s t test). F: Expression of *Bambi* in feather buds of digit 2 is clearly seen by in situ hybridisation on sections (fb: feather buds; pc: perichondrium).
(TIF)

Figure S2 Truncation of digits implies loss of tip and loss of expression of tip markers. Noggin (1 mg/ml, A,C,E) or control PBS (B,D) beads were applied to the tip of digit 1 in the wing or digit 3 in the leg at stages HH 28–31 (a scheme showing the position of the bead in the wing is shown in F). After 4–5 days, embryos were collected and limbs subjected to in situ hybridisation to detect expression of *Bambi* (A–D) or *Sp8* (E). Application of noggin induced loss of the tip and a lack of *Bambi* and *Sp8* expression (arrows in A,C,E). Control beads did not have any effect, and normal tips expressing *Bambi* and *Sp8* were observed (arrowheads in B,D). A,B,E show wings; C, D show legs.
(TIF)

Figure S3 Timing and sequence of phalanx ossification in duck digits. A: Sequence of phalanx ossification visualised by alizarin red and alcian blue staining at the indicated developmental stages. Note that tips (arrowheads at E15) ossify before intermediate phalanges with the sole exception of toe II (arrow, E14). B: Scheme showing the sequence of phalanx ossification in duck digits *Top*: foot, with toes numbered I-IV from anterior to posterior. *Bottom*: wings, with digits numbered 1–3 from anterior to posterior. a: anterior; p: posterior; pr: proximal; d: distal.
(TIF)

Figure S4 Timing and sequence of phalanx ossification in chick digits. Alcian blue and alizarin red stained legs (A) and wings (B) are shown at the indicated days of incubation. A proximodistal ossification sequence is observed, in which the distal phalanx ossifies last, except in the case of toe IV, in which phalange 3 is the last to ossify (the arrow marks the ossifying terminal phalanx in this digit at E13, while the intermediate phalanges have yet to ossify; arrowheads). C: Scheme showing the sequence of phalanx ossification in chick digits. *Top*: foot, with toes numbered I-IV from anterior to posterior. *Bottom*: wing, with digits numbered 1–3 from anterior to posterior. a: anterior; p: posterior; pr: proximal; d: distal.
(TIF)

Figure S5 *Bambi* expression is detected in duck embryos with the chicken probe. Whole mount in situ hybridisation of duck wings and legs at the indicated embryonic stages (E: days of incubation). Expression is seen not only in digit tips but also in feather buds in the wing and scales in the leg (arrows). Note also strong expression in the perichondrium of the humerus (*).
(TIF)

Figure S6 *Sp8* expression in the tip confirms digit transformation after surgical manipulations. Two surgical manipulations were performed in HH27 wings to transform the identity of digit 2 towards digit 1. Seven days after the operation, *Sp8* expression was detected by in situ hybridisation. A: In type I experiments, the posterior part of the digit 2 primordium and the posterior interdigital space 2 were removed (see scheme in the inset). The remnant anterior part of digit 2 primordium has developed into a shorter digit (transformed digit 2*) with a tip positive for *Sp8* expression (arrow). B: In type II experiments the digit 2 primordium was bisected (see scheme in the inset). The extra digit formed from the anterior half of the digit 2 primordium (transformed digit 2*) shows expression of *Sp8* in the tip (arrow). C, D: Additional examples of digit transformation after bisection of digit 2 primordia. Note that the extra digit 2* is shorter, parallel to digit 1, and shows expression of *Sp8* (C) or *Bambi* (D) in the tip (arrows). A, B, D: dorsal views with the operated wing on the right. C shows a ventral view with the operated wing on the left. Note: dark colour of feather buds in B is due to pigment, not to in situ hybridisation signal.
(TIF)

Figure S7 Additional phenotypes obtained after application of Fgf8 beads to the first or second interdigital spaces of the chicken wing (A, fresh specimen, ventral view; B-E, alcian green whole-mount staining, dorsal views). A: Elongation of digit 1 and the presence of a normal tip (arrow) are observed after a bead was implanted in the first ID. B: Alcian green stained wing after Fgf8 bead was applied to the first ID at a slightly earlier time (HH26). Note digit 1 is elongated but fused with digit 2 through an extra cartilage element (arrow). This phenotype is similar to previously reported results. C: In some cases, a nodule of cartilage (arrow) formed in the posterior part of digit 2 after application of an Fgf8 bead to the second ID, but no elongation was apparent. D: An additional example of the effect of Fgf8 bead implanted in the second ID, showing elongation of the digit 3 phalanx (arrow), without induction of a new joint in this case. E: Another example of elongation of digit 3 with the formation of an extra phalanx (arrow) with a new joint (asterisk). F: Scheme showing the position of the beads at the time of operation. Text refers to the panels showing each experiment. Operated wings are shown on the left in panels A,C,D, E and on the right in panel B.
(TIF)

File S1 Time of phalanx ossification in the chick leg (Table 1), chick wing (Table 2), duck leg (Table 3) and duck wing (Table 4).
(PDF)

File S2 Results from manipulations to induce changes of identity in chick wing digit 2. The percentage of embryos showing expression of *Bambi* (Table 5) and *Sp8* (Table 6) in transformed digit tips is shown, along with the numbers of total embryos operated and of each phenotype obtained.
(PDF)
