# Supplementary figures and images for: Bambi and Sp8 Expression Mark Digit Tips and Their Absence Shows That Chick Wing Digits 2 and 3 Are Truncated
Source: PLoS One. 2012 Dec 28;7(12):e52781. doi: 10.1371/journal.pone.0052781 (PMC3532063; doi:10.1371/journal.pone.0052781)

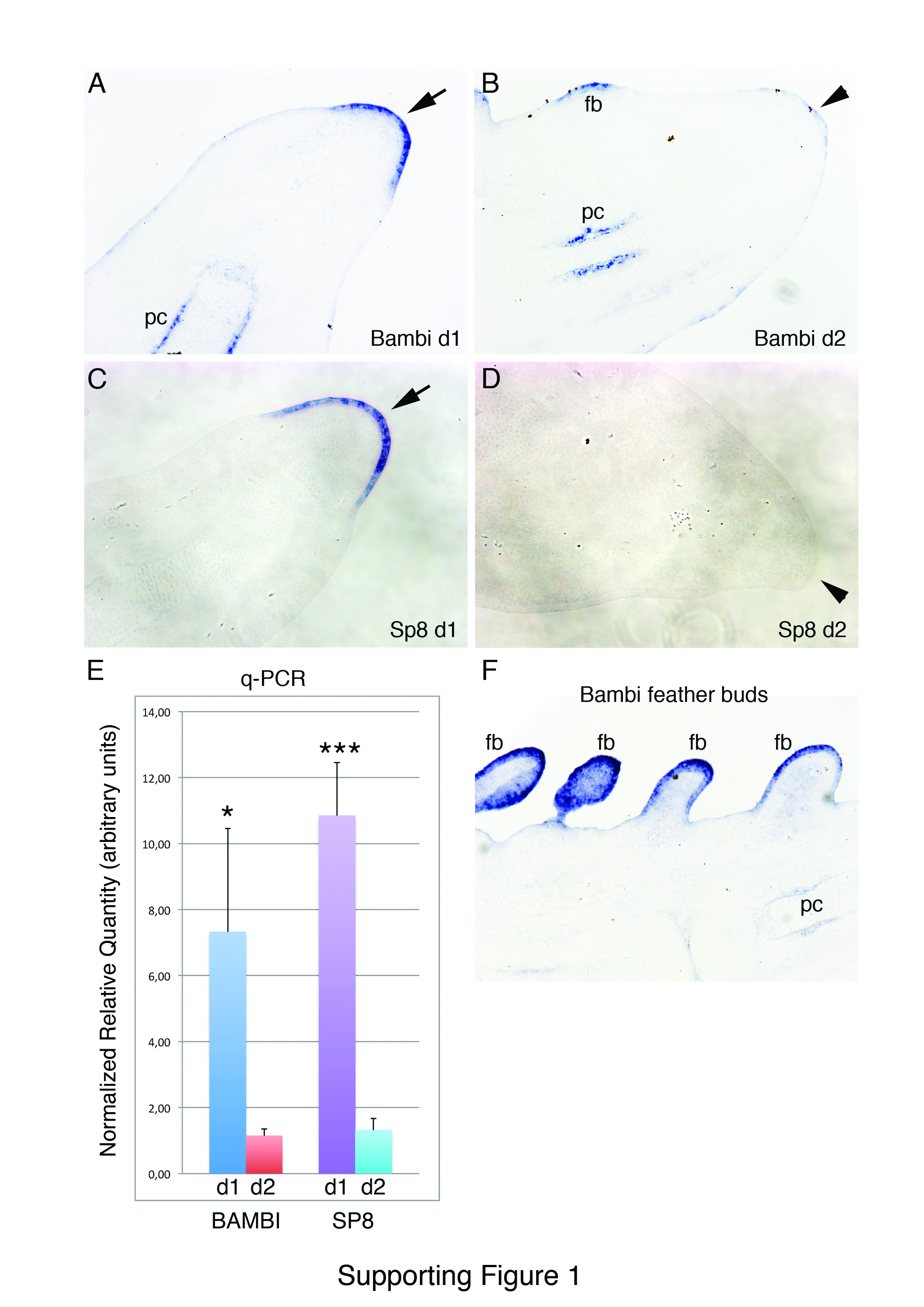

Supplement: Figure S1 — Bambi and Sp8 expression in the chick wing digits 1 and 2 tips. A–D, F. In situ hybridisation on tissue sections from stage HH36 wings. A: Strong expression of Bambi is observed in the digit 1 tip ectoderm (arrow). Expression is also seen in the perichondrium (pc). B: Although Bambi expression is observed in the perichondrium (pc) and feather buds (fb), it is absent from the tip of digit 2 (arrowhead). C: Sp8 is strongly and specifically expressed in the digit 1 tip ectoderm (arrow) but is absent from digit 2 tip (arrowhead in D). E: Real time quantitative PCR (Q-PCR) analysis of the expression of Bambi and Sp8 in digits 1 and 2 tips. A significantly higher expression of both markers is observed in digit 1 tips than in digit 2 tips. Results are given as the normalized relative quantity (+/− SEM) in arbitrary units (*: p<0.05, ***: p<0.001 Student’s t test). F: Expression of Bambi in feather buds of digit 2 is clearly seen by in situ hybridisation on sections (fb: feather buds; pc: perichondrium). (TIF) [file pone.0052781.s001.tif]

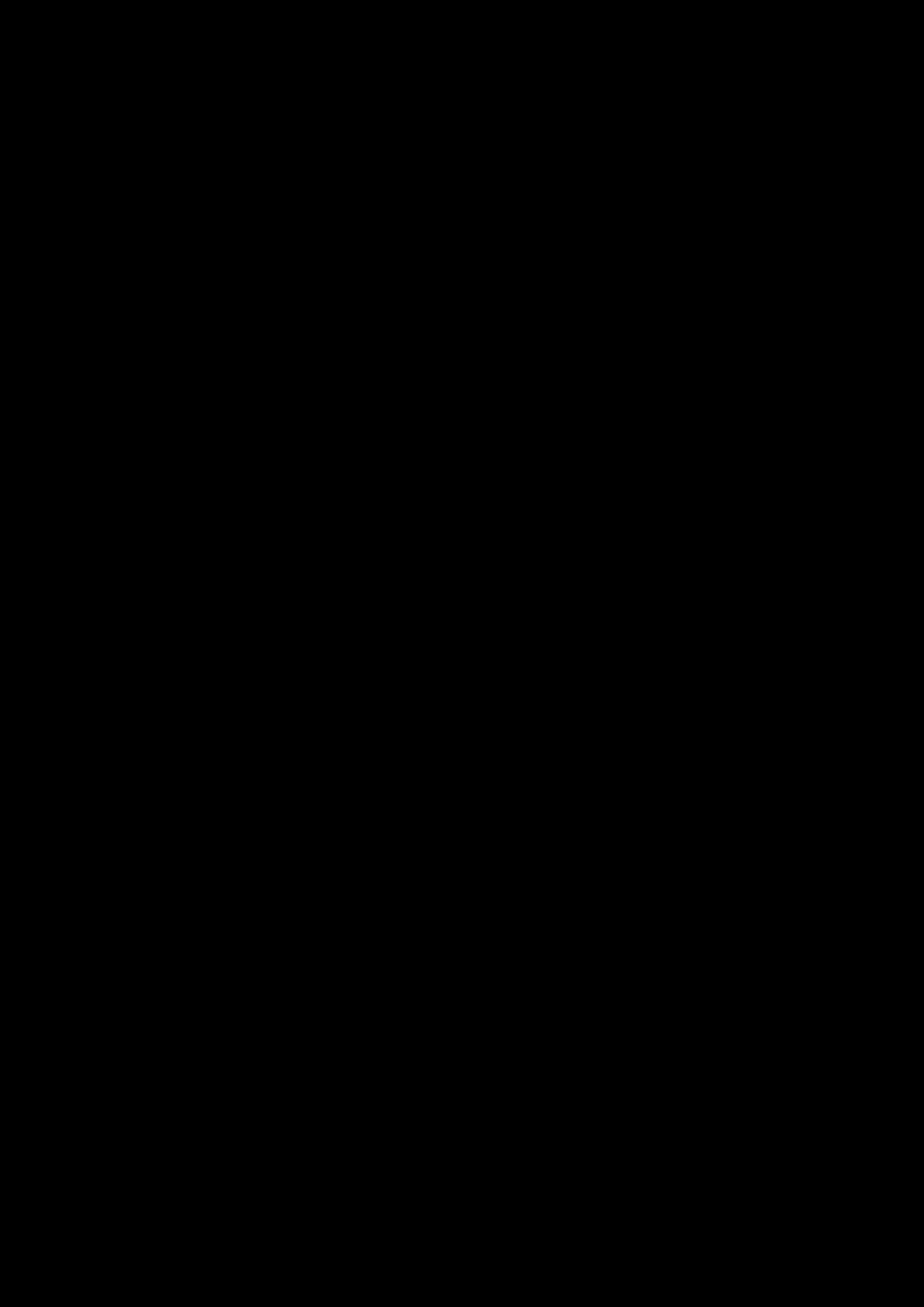

Supplement: Figure S2 — Truncation of digits implies loss of tip and loss of expression of tip markers. Noggin (1 mg/ml, A,C,E) or control PBS (B,D) beads were applied to the tip of digit 1 in the wing or digit 3 in the leg at stages HH 28–31 (a scheme showing the position of the bead in the wing is shown in F). After 4–5 days, embryos were collected and limbs subjected to in situ hybridisation to detect expression of Bambi (A–D) or Sp8 (E). Application of noggin induced loss of the tip and a lack of Bambi and Sp8 expression (arrows in A,C,E). Control beads did not have any effect, and normal tips expressing Bambi and Sp8 were observed (arrowheads in B,D). A,B,E show wings; C, D show legs. (TIF) [file pone.0052781.s002.tif]

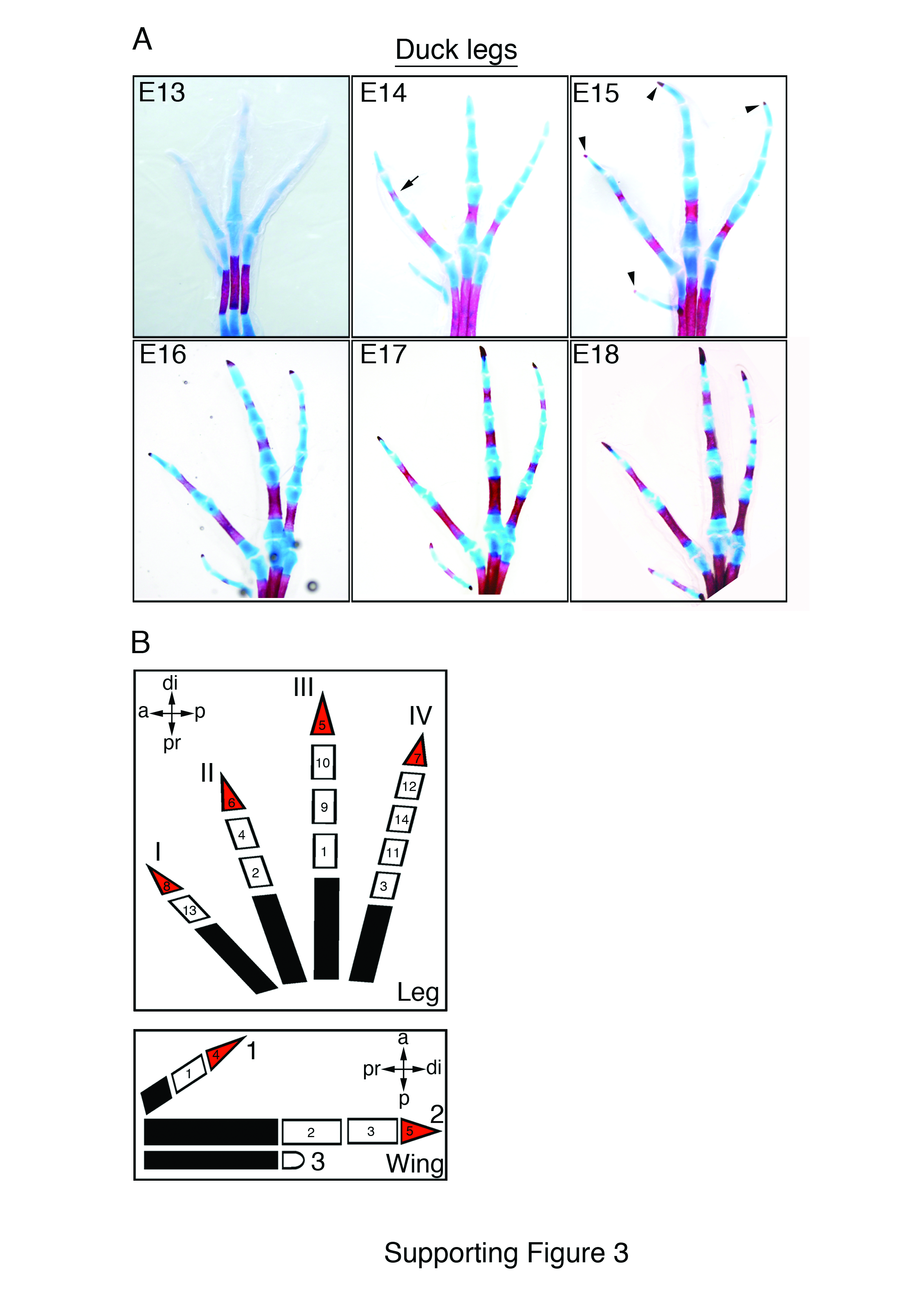

Supplement: Figure S3 — Timing and sequence of phalanx ossification in duck digits. A: Sequence of phalanx ossification visualised by alizarin red and alcian blue staining at the indicated developmental stages. Note that tips (arrowheads at E15) ossify before intermediate phalanges with the sole exception of toe II (arrow, E14). B: Scheme showing the sequence of phalanx ossification in duck digits Top: foot, with toes numbered I-IV from anterior to posterior. Bottom: wings, with digits numbered 1–3 from anterior to posterior. a: anterior; p: posterior; pr: proximal; d: distal. (TIF) [file pone.0052781.s003.tif]

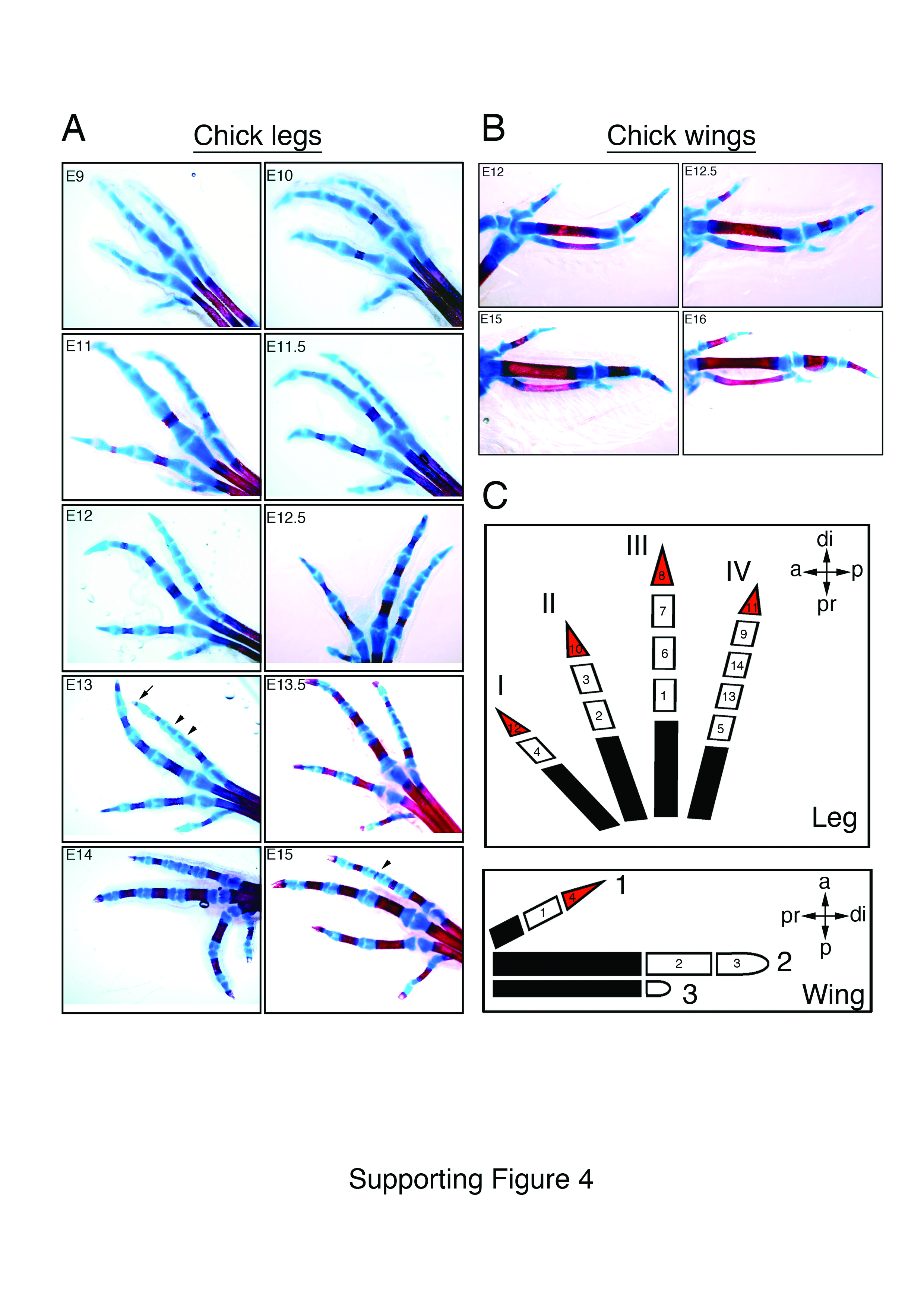

Supplement: Figure S4 — Timing and sequence of phalanx ossification in chick digits. Alcian blue and alizarin red stained legs (A) and wings (B) are shown at the indicated days of incubation. A proximodistal ossification sequence is observed, in which the distal phalanx ossifies last, except in the case of toe IV, in which phalange 3 is the last to ossify (the arrow marks the ossifying terminal phalanx in this digit at E13, while the intermediate phalanges have yet to ossify; arrowheads). C: Scheme showing the sequence of phalanx ossification in chick digits. Top: foot, with toes numbered I-IV from anterior to posterior. Bottom: wing, with digits numbered 1–3 from anterior to posterior. a: anterior; p: posterior; pr: proximal; d: distal. (TIF) [file pone.0052781.s004.tif]

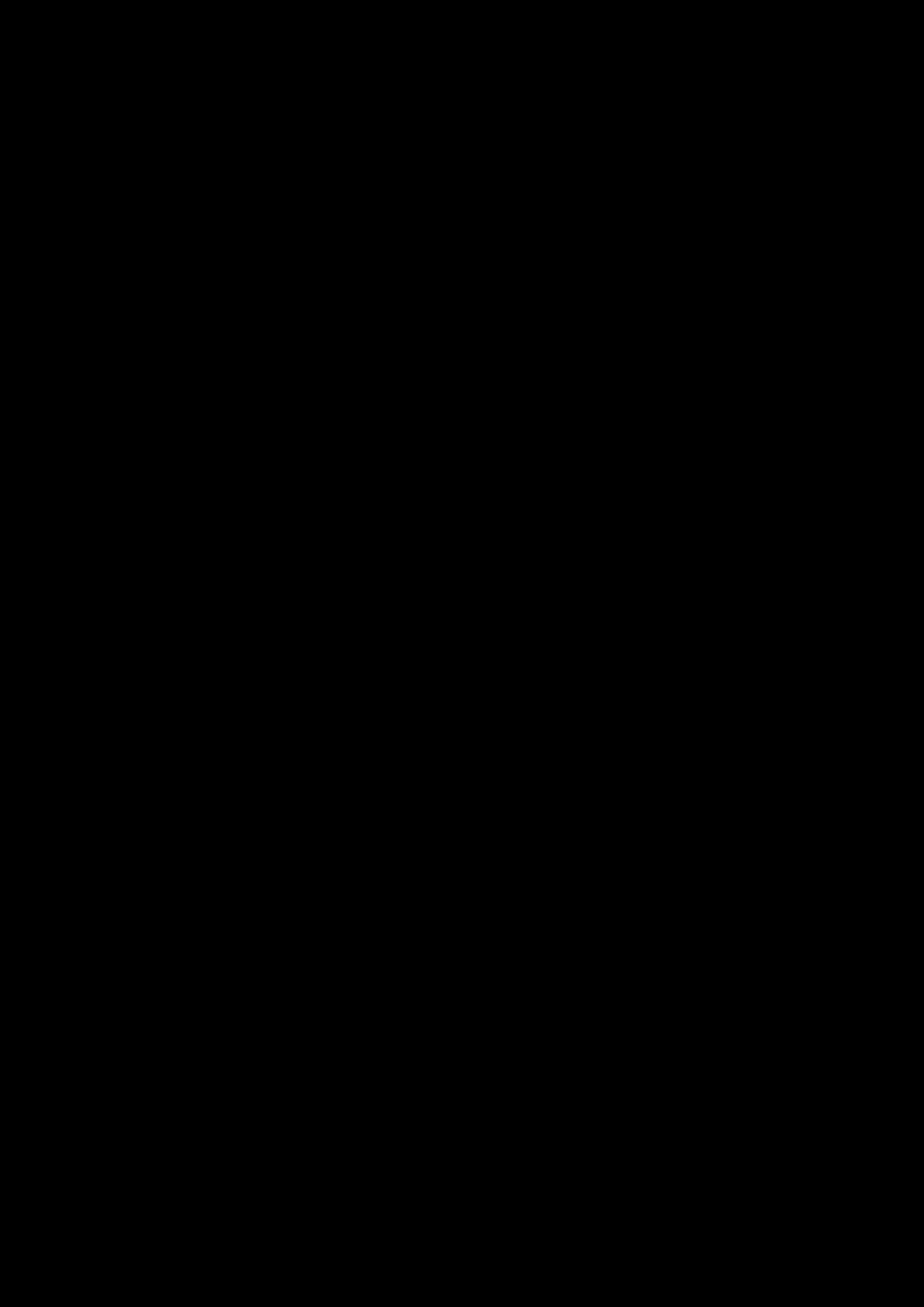

Supplement: Figure S5 — Bambi expression is detected in duck embryos with the chicken probe. Whole mount in situ hybridisation of duck wings and legs at the indicated embryonic stages (E: days of incubation). Expression is seen not only in digit tips but also in feather buds in the wing and scales in the leg (arrows). Note also strong expression in the perichondrium of the humerus (*). (TIF) [file pone.0052781.s005.tif]

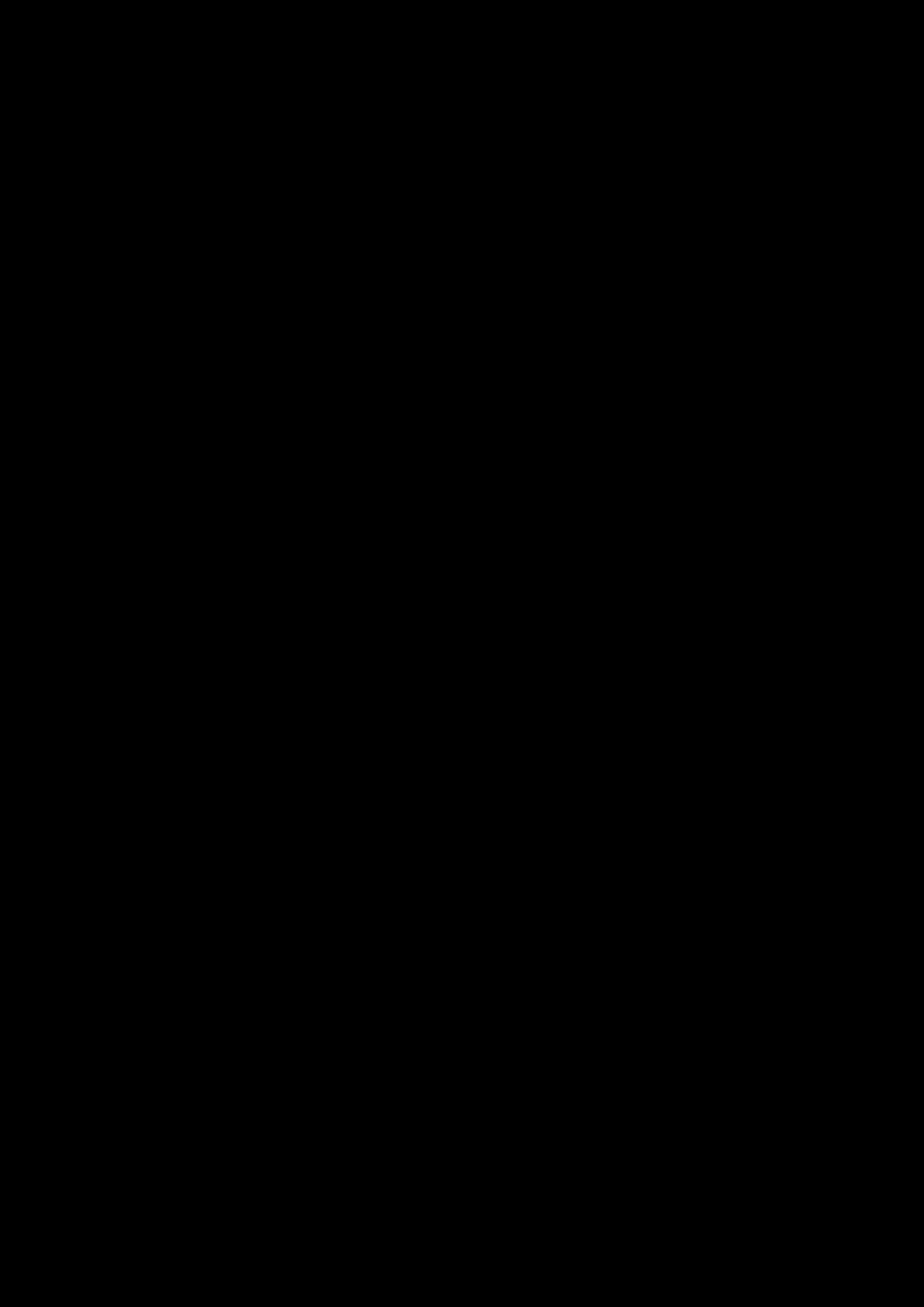

Supplement: Figure S6 — Sp8 expression in the tip confirms digit transformation after surgical manipulations. Two surgical manipulations were performed in HH27 wings to transform the identity of digit 2 towards digit 1. Seven days after the operation, Sp8 expression was detected by in situ hybridisation. A: In type I experiments, the posterior part of the digit 2 primordium and the posterior interdigital space 2 were removed (see scheme in the inset). The remnant anterior part of digit 2 primordium has developed into a shorter digit (transformed digit 2*) with a tip positive for Sp8 expression (arrow). B: In type II experiments the digit 2 primordium was bisected (see scheme in the inset). The extra digit formed from the anterior half of the digit 2 primordium (transformed digit 2*) shows expression of Sp8 in the tip (arrow). C, D: Additional examples of digit transformation after bisection of digit 2 primordia. Note that the extra digit 2* is shorter, parallel to digit 1, and shows expression of Sp8 (C) or Bambi (D) in the tip (arrows). A, B, D: dorsal views with the operated wing on the right. C shows a ventral view with the operated wing on the left. Note: dark colour of feather buds in B is due to pigment, not to in situ hybridisation signal. (TIF) [file pone.0052781.s006.tif]

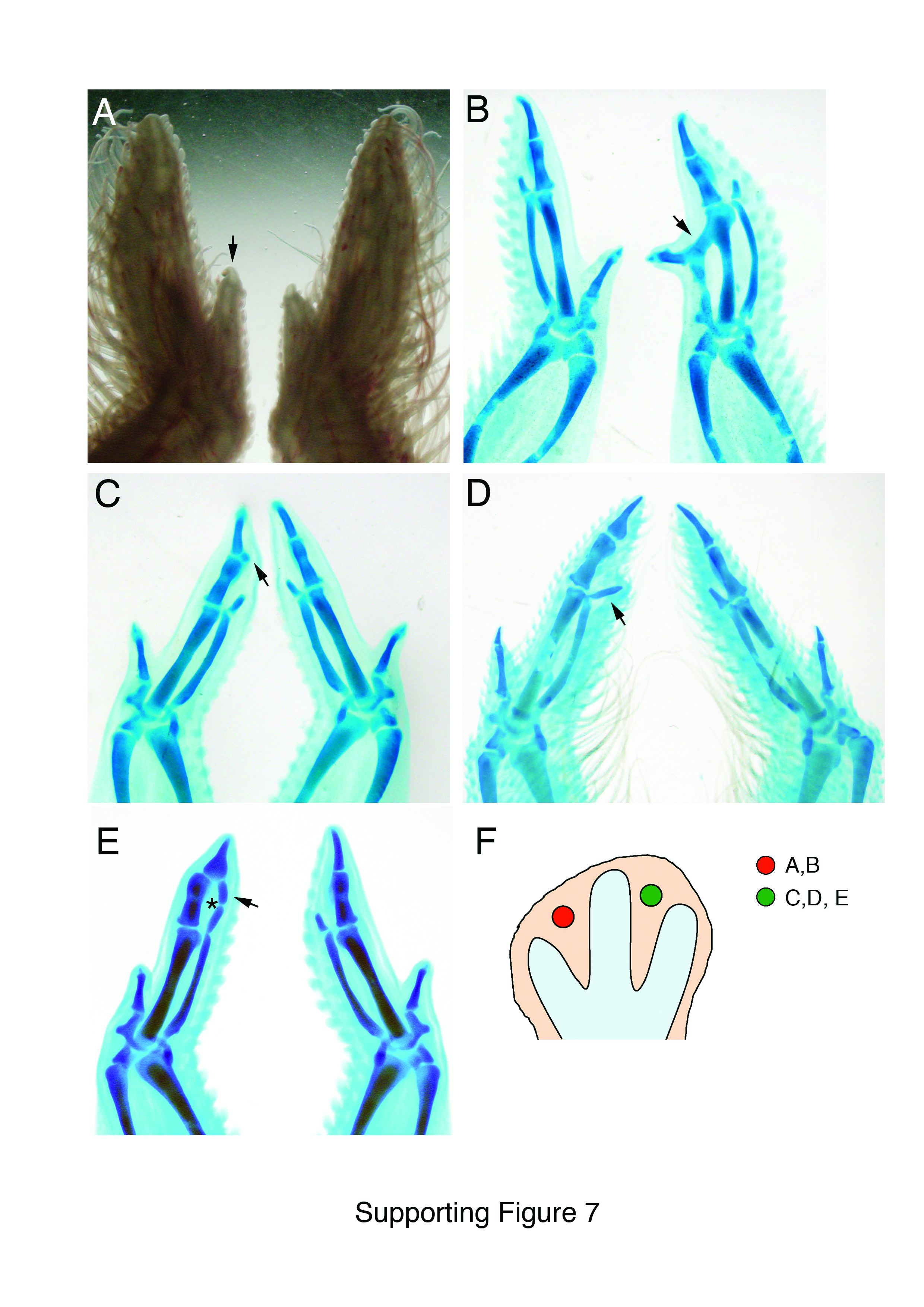

Supplement: Figure S7 — Additional phenotypes obtained after application of Fgf8 beads to the first or second interdigital spaces of the chicken wing (A, fresh specimen, ventral view; B-E, alcian green whole-mount staining, dorsal views). A: Elongation of digit 1 and the presence of a normal tip (arrow) are observed after a bead was implanted in the first ID. B: Alcian green stained wing after Fgf8 bead was applied to the first ID at a slightly earlier time (HH26). Note digit 1 is elongated but fused with digit 2 through an extra cartilage element (arrow). This phenotype is similar to previously reported results. C: In some cases, a nodule of cartilage (arrow) formed in the posterior part of digit 2 after application of an Fgf8 bead to the second ID, but no elongation was apparent. D: An additional example of the effect of Fgf8 bead implanted in the second ID, showing elongation of the digit 3 phalanx (arrow), without induction of a new joint in this case. E: Another example of elongation of digit 3 with the formation of an extra phalanx (arrow) with a new joint (asterisk). F: Scheme showing the position of the beads at the time of operation. Text refers to the panels showing each experiment. Operated wings are shown on the left in panels A,C,D, E and on the right in panel B. (TIF) [file pone.0052781.s007.tif]
