# Supplementary material for: Bambi and Sp8 Expression Mark Digit Tips and Their Absence Shows That Chick Wing Digits 2 and 3 Are Truncated
Source: PLoS One. 2012 Dec 28;7(12):e52781. doi: 10.1371/journal.pone.0052781 (PMC3532063; doi:10.1371/journal.pone.0052781)
Supplement: File S1 — Time of phalanx ossification in the chick leg (Table 1), chick wing (Table 2), duck leg (Table 3) and duck wing (Table 4). (PDF) [file pone.0052781.s008.pdf]

Tables 1-4

Chick

Table 1. Time of phalanx ossification in the chick leg					
**Digit**	**F1**	**F2**	**F3**	**F4**	**F5**
**D1**	11	13-14			
**D2**	10	11	12-13		
**D3**	10	11-12	11-12	12	
**D4**	11	14	15	12-13	13

Table 2. Time of phalanx ossification in the chick wing		
**Digit**	**F1**	**F2**
**D1**	11	14-15
**D2**	11	12
**D3**	no	

Duck

Table 3. Time of phalanx ossification in the duck leg					
**Digit**	**F1**	**F2**	**F3**	**F4**	**F5**
**D1**	15-16	14-15			
**D2**	14	14	14-15		
**D3**	14	15-16	15-16	14-15	
**D4**	14	15-16	17	15-16	14-15

Table 4. Time of phalanx ossification in the duck wing			
**Digit**	**F1**	**F2**	**F3**
**D1**	14	15	
**D2**	14-15	15	16-17
**D3**	no		

Note to Tables 1-4
D1-D4: digits 1 to 4 (anterior to posterior); F1-F5: phalanges 1 to 5 (proximal to distal)
Numbers indicate the incubation day where ossification was first seen
