# Supplementary material for: Bambi and Sp8 Expression Mark Digit Tips and Their Absence Shows That Chick Wing Digits 2 and 3 Are Truncated
Source: PLoS One. 2012 Dec 28;7(12):e52781. doi: 10.1371/journal.pone.0052781 (PMC3532063; doi:10.1371/journal.pone.0052781)
Supplement: File S2 — Results from manipulations to induce changes of identity in chick wing digit 2. The percentage of embryos showing expression of Bambi (Table 5) and Sp8 (Table 6) in transformed digit tips is shown, along with the numbers of total embryos operated and of each phenotype obtained. (PDF) [file pone.0052781.s009.pdf]

Table 5: Expression of *Bambi* in transformed digits

Table 5: Manipulations to induce change of identity in chick wing digit 2					
Number of embryos >>>>	Total Collected	Normal appearance	Abnormal but not obvious transformation of digit identity	Morphological transformation observed	*Bambi* expression present in transformed D2* tip
Type I Experiments: <u>Removal</u> of postP2+pID2	84	9 (11% of total)	21 (25% of total)	54 (64% of total)	8 (15% of transformed, 10% of collected)
Type II Experiments: <u>Bisection</u> of P2	38	8 (21% of total)	12 (32% of total)	18 (47% of total)	6 (33% of transformed, 16% of collected)

Table 6: Expression of *Sp8* in transformed digits

Table 6: Manipulations to induce change of identity in chick wing digit 2					
Number of embryos >>>>	Total Collected	Normal appearance	Abnormal but not obvious transformation of digit identity	Morphological transformation observed	*Sp8* expression present in transformed D2* tip
Type I Experiments: <u>Removal</u> of postP2+pID2	11	3 (27% of total)	2 (18% of total)	6 (55% of total)	1 (17% of transformed, 9% of collected)
Type II Experiments: <u>Bisection</u> of P2	33	6 (18% of total)	16 (48% of total)	11 (33% of total)	3 (27% of transformed, 9% of collected)
